# Large squamous cell carcinoma of the lip in a Black woman with a history of hydrochlorothiazide use treated with Mohs micrographic surgery

**DOI:** 10.1016/j.jdcr.2023.05.021

**Published:** 2023-05-26

**Authors:** Austinn C. Miller, Vladimir Ratushny, Andras Schaffer, Armand B. Cognetta

**Affiliations:** a Dermatology Associates of Tallahassee, Tallahassee, Florida; b University of Central Florida/HCA Consortium, Tallahassee, Florida; c Division of Dermatology, Mohs Micrographic Surgery Unit, Florida State University College of Medicine, Tallahassee, Florida

**Keywords:** 40-gene expression profile, 40-GEP, HCTZ, hydrochlorothiazide, lip, Mohs micrographic surgery, SCC, squamous cell carcinoma

## Introduction

Squamous cell carcinoma (SCC) of the lip comprises 25% to 30% of all oral cancers, with an estimated incidence of 0.4 in 100,000 per year.^1^ Multiple studies have demonstrated that SCC is the most common type of nonmelanoma skin cancer (NMSC) to affect the vermilion lip, although some studies have suggested that basal cell carcinoma is more common.^1^, ^2^, ^3^, ^4^, ^5^ However, it is widely accepted that SCC occurs more commonly on the lower vermilion lip, accounting for ∼80% of NMSC cases in this region.^2^^,^^4^

SCC of the lip frequently begins as actinic cheilitis, a premalignant ulcerative lesion, and is often detected at an early stage owing to its highly visible location and slow growth pattern.^1^ Several risk factors have been associated with lip SCC, including male sex, older age, fair skin, and sun exposure.^1^ As such, female sex and dark skin are protective factors.^1^^,^^6^ The largest study of lip SCC to date analyzed 15,832 cases, noting that the incidence of SCC in female and Black patients is 18.2% and 0.7%, respectively.^1^

Herein, we present a case of a large exophytic SCC of the lower vermilion lip in a Black female patient with a history of hydrochlorothiazide use treated with Mohs micrographic surgery (MMS). Consent was provided by the patient for publication of case details and images.

## Case report

A 75-year-old Black woman presented with a large mass on her lower lip. The mass had been present for 6 months and had rapid growth. She denied any associated symptoms aside from tenderness to palpation and occasional minor bleeding secondary to dryness and fissuring of the lower lip. The patient reported that she was otherwise in good health, noting a history of well-controlled hypertension treated with ≥12.5 mg of hydrochlorothiazide (HCTZ) for >10 years.

**Fig 1:**
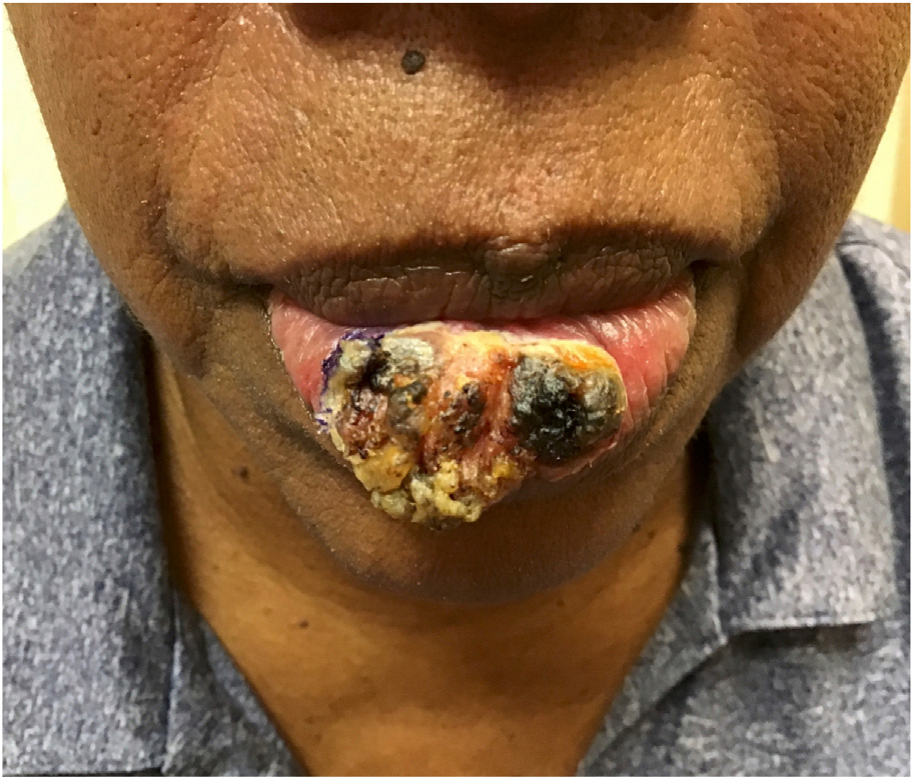
Large exophytic squamous cell carcinoma confined to lower vermilion lip.

On examination, a large, 3.5 × 2.5-cm^2^ exophytic tumor with hyperkeratotic scales was found to be located on the lower vermilion lip, spanning at least 70% of its length (Fig 1). There was no obvious extension to the oral cavity or cutaneous lip. There was no palpable lymphadenopathy throughout.

A shave biopsy was performed, demonstrating invasive SCC. Treatment options for tumor debulking were discussed, and the patient elected for excision with MMS.

The patient underwent 2 Mohs stages to achieve clear margins, with a final wound defect of 4.6 × 2.2 cm^2^. Although frozen sections did not reveal perineural involvement, further evaluation of permanent sections of the debulking layer demonstrated small-caliber perineural involvement (<0.1 mm). Given the tumor size and perineural involvement, she was staged at T2b. Forty-gene expression profile testing was indicated given high-risk tumor factors. It demonstrated a class 2A result, signifying an 80.5% 3-year metastasis free survival rate. She was referred to radiation oncology for adjuvant radiation therapy of the lip.

## Discussion

Although SCC is a relatively common cancer of the lip, large tumors are rare. A review of 581 cases of lip NMSC revealed that only 9.4% of SCCs are >3 cm, with the largest reported to be between 6 and 7.9 cm.^5^ One extraordinary report described a 10-cm tumor.^7^ MMS is an appropriate treatment option for SCC of the lip, no matter the size, with excellent clearance and low rates of recurrence. Leibovitch et al^5^ followed up 23 cases of SCC for 5 years after MMS excision and demonstrated no recurrence. Furthermore, Mohs and Snow^8^ showed a 94.3% cure rate in 1119 patients with lip SCC who completed a 5-year follow-up status after MMS.

The overall prognosis for lip SCC is favorable, with a ∼90% 5-year survival rate across numerous studies.^1^^,^^9^ Age, primary site, T stage, and N stage are determinants of overall survival.^1^ Several risk factors have been associated with lip SCC, including male sex, older age, fair skin, tobacco use, and sun exposure.^1^^,^^10^ In the authors’ experience, HCTZ has been associated with an increased risk and higher burden of NMSC, especially when no other risk factors are present.^11^ A large Danish case-control study is corroborating, demonstrating an adjusted odds ratio for development of lip SCC of 2.1 in those with ever use of HCTZ.^12^ There was a clear dose-response effect, with higher cumulative doses significantly increasing the odds ratio.^12^ An estimated 11% of 633 cases were thought to be HCTZ induced.^12^ Similar studies have mirrored these results, demonstrating an increased risk of nonlip NMSC associated with HCTZ use.^13^ No association was seen with the use of other diuretics or nondiuretic antihypertensives, suggesting that the use of other antihypertensives, when possible, would be prudent.^12^^,^^13^

Black women appear to be the least common group to present with lip SCC, accounting for ∼1 in 1000 lip SCC cases.^1^ Some factors contributing to this lower incidence may be darker pigmentation and lower frequency of human papillomavirus among Blacks.^14^ Despite the lower incidence of SCC among Blacks, they present with more aggressive, late-stage tumors and have worse survival outcomes. Multifactorial explanations have been proposed to explain these poorer outcomes, including differences in tumor biology, later presentation, treatment differences, socioeconomic status, and residential segregation.^15^^,^^16^ Eliminating disparities and looking more closely at each cause will permit opportunities for intervention and better outcomes for lip SCC.

## Conflicts of interest

None disclosed.

## References

[1] Han A.Y., Kuan E.C., Mallen-St Clair J., Alonso J.E., Arshi A., St John M.A. (2016). Epidemiology of squamous cell carcinoma of the lip in the United States: a population-based cohort analysis. JAMA Otolaryngol Head Neck Surg.

[2] Queen D., Knackstedt T., Polacco M.A., Collins L.K., Lee K., Samie F.H. (2019). Characteristics of non-melanoma skin cancers of the cutaneous perioral and vermilion lip treated by Mohs micrographic surgery. J Eur Acad Dermatol Venereol.

[3] Alves A.M., Correa M.B., Silva K.D. (2017). Demographic and clinical profile of oral squamous cell carcinoma from a service-based population. Braz Dent J.

[4] Dawn A., Lawrence N. (2013). Significant differences in nonmelanoma skin cancers of the upper and lower lip. Dermatol Surg.

[5] Leibovitch I., Huilgol S.C., Selva D., Paver R., Richards S. (2005). Cutaneous lip tumours treated with Mohs micrographic surgery: clinical features and surgical outcome. Br J Dermatol.

[6] Pogoda J.M., Preston-Martin S. (1996). Solar radiation, lip protection, and lip cancer risk in Los Angeles County women (California, United States). Cancer Causes Control.

[7] Sheen Y.T., Chen Y.Y., Sheen M.C. (2020). Case report of a huge lower lip cancer successfully treated with intra-arterial infusion chemotherapy. Int J Surg Case Rep.

[8] Mohs F.E., Snow S.N. (1985). Microscopically controlled surgical treatment for squamous cell carcinoma of the lower lip. Surg Gynecol Obstet.

[9] Bhandari K., Wang D.S., Li S.C. (2015). Primary cN0 lip squamous cell carcinoma and elective neck dissection: systematic review and meta-analysis. Head Neck.

[10] Czerninski R., Zini A., Sgan-Cohen H.D. (2010). Lip cancer: incidence, trends, histology and survival: 1970-2006. Br J Dermatol.

[11] Han S., Wolfe C.M., Angnardo L. (2020). Hydrochlorothiazide use and increased squamous cell carcinoma burden in a high-risk Mohs population: a cross-sectional study. Dermatol Surg.

[12] Pottegård A., Hallas J., Olesen M. (2017). Hydrochlorothiazide use is strongly associated with risk of lip cancer. J Intern Med.

[13] Pedersen S.A., Gaist D., Schmidt S.A.J., Hölmich L.R., Friis S., Pottegård A. (2018). Hydrochlorothiazide use and risk of nonmelanoma skin cancer: a nationwide case-control study from Denmark. J Am Acad Dermatol.

[14] Peterson C.E., Khosla S., Chen L.F. (2016). Racial differences in head and neck squamous cell carcinomas among non-Hispanic black and white males identified through the National Cancer Database (1998-2012). J Cancer Res Clin Oncol.

[15] Harris J.A., Hunter W.P., Ji Y.D., Hanna G.J. (2021). Effects of racial residential segregation on oral squamous cell carcinoma prognosis and survival. Oral Oncol.

[16] Carroll W.R., Kohler C.L., Carter V.L., Hannon L., Skipper J.B., Rosenthal E.L. (2009). Barriers to early detection and treatment of head and neck squamous cell carcinoma in African American men. Head Neck.

